# Characteristics, Process Parameters, and Inner Components of Anaerobic Bioreactors

**DOI:** 10.1155/2014/841573

**Published:** 2014-01-23

**Authors:** Awad Abdelgadir, Xiaoguang Chen, Jianshe Liu, Xuehui Xie, Jian Zhang, Kai Zhang, Heng Wang, Na Liu

**Affiliations:** ^1^State Environmental Protection Engineering Center for Pollution Control in Textile, College of Environmental Science and Engineering, Donghua University, Shanghai 201620, China; ^2^Industrial Research and Consultancy Center (IRCC), Khartoum 13314, Sudan

## Abstract

The anaerobic bioreactor applies the principles of biotechnology and microbiology, and nowadays it has been used widely in the wastewater treatment plants due to their high efficiency, low energy use, and green energy generation. Advantages and disadvantages of anaerobic process were shown, and three main characteristics of anaerobic bioreactor (AB), namely, inhomogeneous system, time instability, and space instability were also discussed in this work. For high efficiency of wastewater treatment, the process parameters of anaerobic digestion, such as temperature, pH, Hydraulic retention time (HRT), Organic Loading Rate (OLR), and sludge retention time (SRT) were introduced to take into account the optimum conditions for living, growth, and multiplication of bacteria. The inner components, which can improve SRT, and even enhance mass transfer, were also explained and have been divided into transverse inner components, longitudinal inner components, and biofilm-packing material. At last, the newly developed special inner components were discussed and found more efficient and productive.

## 1. Introduction 

Anaerobic digestion (AD) is an attractive option for waste treatment practice in which both energy recovery and pollution control can be achieved. Anaerobic degradation or digestion involves the breakdown of biomass by a concerted action of a wide range of microorganisms in the absence of oxygen. The biological processes are essentially used to remove contaminants, in wastewater treatment, and nowadays many biological treatment options are available and have shown encouraging results over the treatment of complex organic matter [1, 2]. Comparing between anaerobic and aerobic process, anaerobic process is especially considered as a suitable treatment option due to low-energy requirements and little quantities of sludge production. Therefore, anaerobic process has become increasingly demanding in the treatment of complex industrial wastewater, which may contain toxic materials, or even low concentrations of domestic wastewater [3, 4]. The ability to attain environmental protection and resource preservation, anaerobic treatment process, and anaerobic bioreactors has received great attention [3, 5, 6].

The anaerobic digestion process is a simple and applied energy source. A simple digester consists of a digestion chamber, a dome, an inlet, an outlet for biogas, and an outlet for slurry. The biogas trapped by the dome flows under pressure through the outlet, where it can be used as an energy source. The use of anaerobic reactor began in 1859 with the first anaerobic digester that was built by a leper colony in Bombay, India [7]. And then in 1895, the technology was developed in Exeter, England, where a septic tank was used to generate gas for the sewer gas destructor lamp, a type of gas lighting. Thereafter, in the 1930s, anaerobic digestion gained academic recognition through scientific research [8]. The septic tank represents the first generation of anaerobic bioreactor (AB). In the late 1970s, the upflow anaerobic sludge blanket (UASB) process was developed by Dr. Gatze Lettinga and colleagues to represent the second generation of AB [9, 10]. In the beginning, a UASB reactor was just like an empty tank (thus an extremely simple and inexpensive design), but an important inner component, the three-phase separator, is added to avoid washout of granular sludge occurred. The third generation of AB, such as expanded granular sludge bed (EGSB) and internal circulation (IC) reactor, was developed on the foundation of the second generation. For instance, the faster rate of upward-flow velocity is designed for the wastewater passing through the sludge bed. The increased flux permits partial expansion (fluidization) of the granular sludge bed, improving wastewater-sludge contact as well as enhancing segregation of small inactive suspended particle from the sludge bed. The increased flow velocity is either accomplished by utilizing tall reactors or by incorporating an effluent recycle (or both) [3, 11–13]. Some super-high-rate anaerobic bioreactors (SAB) were developed recently by Chen et al. [11–13]. The development of the third generation anaerobic bioreactors and SAB shows that the process parameters such as upflow velocity, Sludge Retention Time (SRT) and Hydraulic Retention Time (HRT); and bioreactor structure (inner components) are the two most important factors for microorganisms' cultivation for anaerobic bioreactors [3, 11–13]. Therefore, This work will present the advantages and disadvantages of anaerobic process and explain the representative characteristics of anaerobic bioreactor. It also introduces main process parameters, inner components, and their effects on the performance of wastewater treatment in anaerobic bioreactors.

## 2. Advantages and Disadvantages of Anaerobic Process

Anaerobic treatment (digestion) is a proven way and efficient method to produce biogas (methane) that can be used for the production of renewable heat and power and a compost like output. The principle of anaerobic treatment is the utilization of anaerobic bacteria (biomass) to convert organic matter (pollutants) or COD (chemical oxygen demand) into methane rich biogas in the absence of oxygen.

The early stages of the formation of anaerobic granules follow the same principles in the formation of biofilm of bacteria on solid surfaces. There is strong evidence that inert carriers play an important positive role in granulation. Many researchers conclude that *Methanosaeta concilii* is a key organism in granulation [14].

When the system is in balance, the methanogenic bacteria use the acid intermediates as rapidly as they appear. However, if the methane bacteria are not present in suitable numbers or are being slowed down by unfavorable environmental conditions, they will not use the acids as rapidly as they are produced by the acid formers, and the volatile acids will increase in concentration. Thus, an increase in acid concentration indicates that the methane formers are not in balance with the acid formers [5]. Classification of bacteria depends upon temperature classes, where mesophiles bacteria are like mesophilic temperature, while thermophiles bacteria are like thermophilic temperature. Advantages and disadvantages of anaerobic treatment processes are shown in Table 1.

## 3. Characteristics of Anaerobic Bioreactor

### 3.1. Inhomogeneous System

Anaerobic reactor operates under an inhomogeneous system that means that the anaerobic treatment is done through three phases, solid (sludge), liquid (wastewater), and gas (methane).

#### 3.1.1. Solid Phase

Solid phase consists of sludge granules, which have a diameter around 0.5 to 2 mm [14], which exist in the lower part of the reactor. Sludge granules play an important role for successful operation of UASB and EGSB technology, a sludge granules are the sum of microorganisms (Bacteria) forming during the treatment of wastewater in an environment with a constant upflow hydraulic regime, without any support matrix, the surrounding and suitable environment for bacteria survive is occurring during flow conditions, and therefore bacteria able to contact together and start growth and proliferate.

#### 3.1.2. Liquid Phase

Liquid (wastewater) flows upwards through a sludge bed located in the lower part of the reactor, while the upper part contains a three phases (solid, liquid, and gas) of separation system. Three-phase separation device is the most characteristic feature of UASB reactor. It facilitates the collection of biogas and also provides internal recycling of sludge by disengaging adherent biogas bubbles from rising sludge particles.

#### 3.1.3. Gas Phase

Biogas typically refers to a gas produced by bacteria that breakdown organic matter in the absence of oxygen (organic waste such as dead plant and animal material, animal feces, and kitchen waste or industrial organic waste can be converted into a gaseous fuel called biogas). The most important thing is that biogas can result in the dynamic behavior of granular sludge [13].

**Figure 1:**
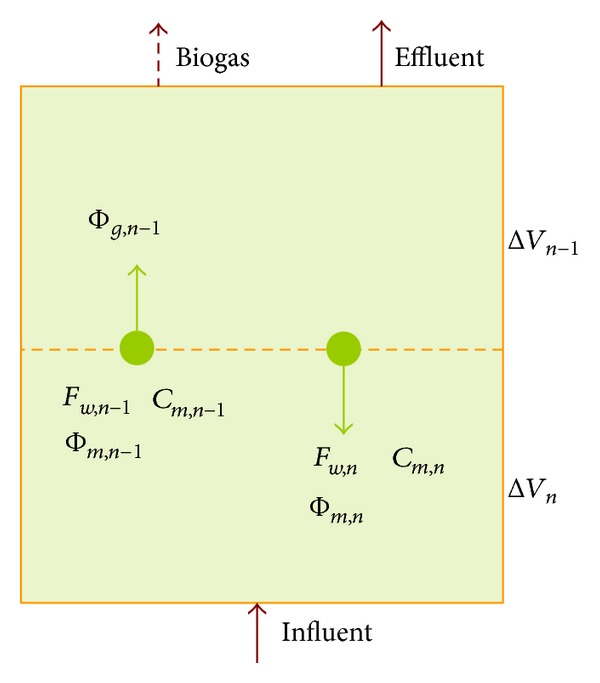
The granular sludge dynamic behavior.

In Figure 1, *ϕ*_*g*,*n*−1_ is gas production in Δ*V*_*n*−1_, m^3 ^h^−1^, *ϕ*_*m*,*n*_ is downwards transport of sludge from Δ*V*_*n*_ to Δ*V*_*n*−1_, kg h^−1^, *ϕ*_*m*,*n*−1_ is sludge transport flux from Δ*V*_*n*−1_ to Δ*V*_*n*_, kg h^−1^, *C*_*m*,*n*_ is sludge concentration (TSS) in Δ*V*_*n*_, kg m^−3^, *C*_*m*,*n*−1_ is sludge concentration (TSS) in Δ*V*_*n*−1_, kg m^−3^, *F*_*w*,*n*−1_ is up-moving wake stream in Δ*V*_*n*−1_, m^3^ h^−1^, and *F*_*w*,*n*_ is backmix stream in Δ*V*_*n*_, m^3^ h^−1^.

Figure 1 explains the relationships that happen, while the movements of sludge up and down due to gas production, and therefore the transport power of sludge resulted from the upward fluid flow led by influence and the up-moving wake stream (*F*_*w*,*n*−1_, m^3^ h^−1^) led by gas production (*ϕ*_*g*,*n*−1_). Buijs et al. (1982) reported that the sludge transport efficiency of up-moving biogas can reach up to 20.2 ± 2.1 m^3^/m^3^ (sludge/biogas) in a UASB reactor [16]. Figure 1 suggested that the downward transport of sludge (*ϕ*_*m*,*n*_, Kg h^−1^) from Δ*V*_*n*_ to Δ*V*_*n*−1_ was caused due to the settling of sludge and by the back mix stream [13].

### 3.2. Time Instability

Anaerobic Bioreactor is used for treatment of wastewater, consisting of complex organic matters. It is become more complicated with the change in production cycles and quantities. Various industrial processes operating under different conditions (times, temperature, and pH) with a different range, throughout the four seasons of the year, have created a challenge on AB. Due to the inherent limitations of anaerobic treatment technologies, there is a need to focus on the improvement of these drawbacks, thus challenging the designers and engineers [6].

Many small sewage treatment plants are operated independently in local cities, due to encouraging processing by anaerobic digestion at a centralized sewage treatment plant (STP); high-solid sewage sludge is helpful because it reduces the energy and cost required for transporting the sludge from other STPs. Hidaka et al. [17] reported that under mesophilic condition, anaerobic digestion of sewage sludge of total solids concentrations (TS) of 10% was successfully treated, while under the thermophilic condition, sewage sludge of 7.5% TS was not successfully treated when the total ammonia concentration was over 2000 mg N/L. But thermophilic anaerobic digestion successfully reduced *Salmonella *spp., and *Escherichia coli* is below detection limits but not *Clostridium perfringens* Spores. Thus, the final product met Class A biosolids' final disposal regulations, but further investigation is needed in order to satisfy the future European legislation [18].

### 3.3. Space Instability

**Figure 2:**
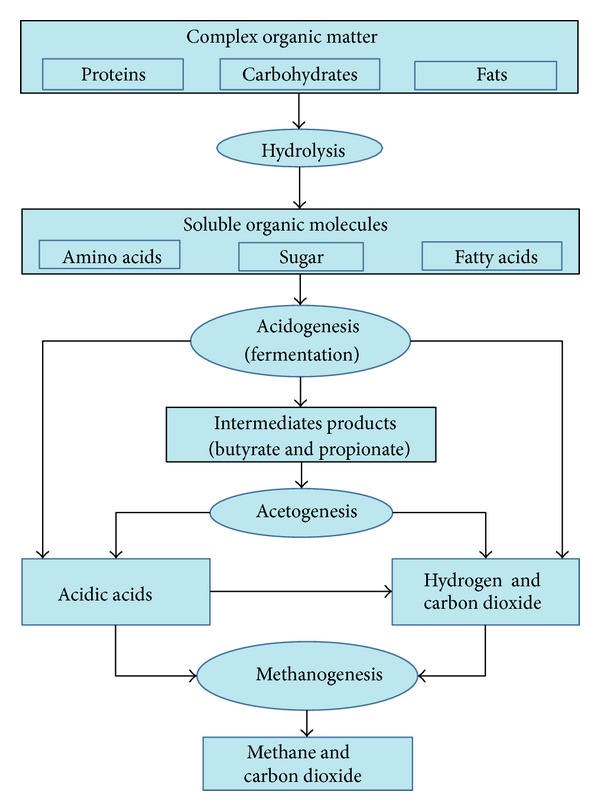
The key process stages of anaerobic digestion.

The microbial consortia which achieve the conversion of complex organic matter into biogas are formed of several groups and each performs a specific function in the digestion process. Together they achieve the conversion of organic matter into biogas through a sequence of stages. The initial stages generate short chain or volatile fatty acids (VFA). The final stage is methanogenesis (methane formation) where methanogenic precursors, primarily acetic acid and hydrogen, are converted to methane and CO_2_.  Figure 2 explains the key process stages of anaerobic digestion [19].

The first stage of digestion process is hydrolysis. This step is very important for the anaerobic digestion process since polymers cannot be directly utilized by the fermentative microorganisms. Therefore, the insoluble complex organic matter, such as cellulose, converts into soluble molecules such as sugars, amino acids, and fatty acids. The complex polymeric matter is hydrolyzed to monomer, for example, cellulose to sugars or alcohols and proteins to peptides or amino acids, by hydrolytic enzymes (lipases, proteases, cellulases, amylases, etc.), secreted by microbes, and make them available for other bacteria [20, 21]. Equation (1) shows an example of a hydrolysis reaction where a polysaccharide is broken down into glucose [20, 21].

Hydrolysis reactions is as follows:

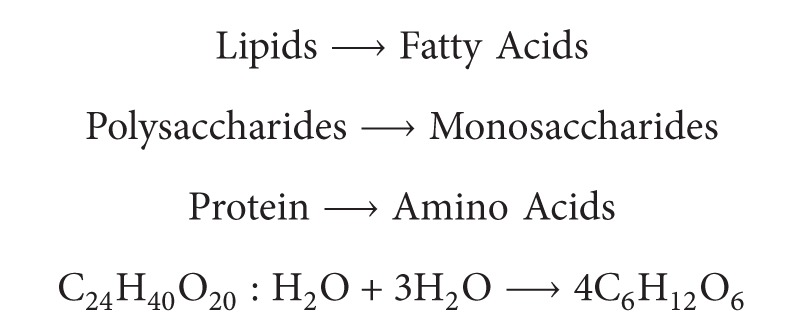
(1)


In the second stage acidogenesis (fermentation), acidogenic bacteria transform the products of the first reaction (such as sugars and amino acids) into carbon dioxide, hydrogen, ammonia, and organic acids. The principal acidogenesis stage products are acetic acid (CH_3_COOH), propionic acid (CH_3_CH_2_COOH), butyric acid (CH_3_CH_2_CH_2_COOH), and ethanol (C_2_H_5_OH). In an equilibrated system, most of the organic matter is converted into readily available substrates for methanogenic microbes (acetate, hydrogen, and carbon dioxide), but a significant part (approximately 30%) is transformed into short chain fatty acids or alcohols [15]. From these products, the hydrogen, carbon dioxide, and acetic acid will skip the third stage (acetogenesis) and be utilized directly by the methanogenic bacteria in the final stage (methanogenesis). Equations (2), (3) [22], and (4) [15] represent three typical acidogenesis reactions where glucose is converted to ethanol, propionate, and acetic acid, respectively,

(2) $$\begin{array}{c} {\text{C}}_{6}{\text{H}}_{12}{\text{O}}_{6}⟶2\text{C}{\text{H}}_{3}\text{C}{\text{H}}_{2}\text{OH}+2\text{C}{\text{O}}_{2} \end{array}$$

(3) $$\begin{array}{c} {\text{C}}_{6}{\text{H}}_{12}{\text{O}}_{6}+2{\text{H}}_{2}⟶2\text{C}{\text{H}}_{3}\text{C}{\text{H}}_{2}\text{COOH}+2{\text{H}}_{2}\text{O} \end{array}$$

(4) $$\begin{array}{c} {\text{C}}_{6}{\text{H}}_{12}{\text{O}}_{6}+2{\text{H}}_{2}\text{O}⟶2\text{C}{\text{H}}_{3}\text{COOH}+2\text{C}{\text{O}}_{2}+4{\text{H}}_{2} \end{array}$$

In the third stage, acetogenic bacteria convert the organic acids that resulting from the second stage and the rest of the acidogenesis products into acetic acid, hydrogen, and carbon dioxide. Equation (5) represents the conversion of propionate to acetate, only achievable at low hydrogen pressure. Glucose (6) and ethanol (7) among others are also converted to acetate during the third stage of anaerobic digestion process [22]. The products formed during acetogenesis are due to a number of different microbes, for example, *syntrophobacter wolinii*, *a propionate decomposer *and* sytrophomonos wolfei, a butyrate decomposer*. Other acid formers are *Clostridium* spp.,* Peptococcus anaerobius, Lactobacillus, *and *Actinomyces *[15],

(5) CH3CH2COO−+3H2O ⟶CH3COO−+H++HCO3−+3H2

(6) $$\begin{array}{c} {\text{C}}_{6}{\text{H}}_{12}{\text{O}}_{6}+2{\text{H}}_{2}\text{O}⟶2\text{C}{\text{H}}_{3}\text{COOH}+2\text{C}{\text{O}}_{2}+4{\text{H}}_{2} \end{array}$$

(7) $$\begin{array}{c} \text{C}{\text{H}}_{3}\text{C}{\text{H}}_{2}\text{OH}+2{\text{H}}_{2}\text{O}⟶\text{C}{\text{H}}_{3}\text{CO}{\text{O}}^{-}+2{\text{H}}_{2}+{\text{H}}^{+} \end{array}$$

The fourth and final stage is called methanogenesis. In this stage, methane is produced by bacteria called methanogens (also known as methane former) in two ways: either by means of cleavage of acetic acid molecules to generate carbon dioxide and methane (8) or by reduction of carbon dioxide with hydrogen (9). Methane production is higher from reduction of carbon dioxide, but limited hydrogen concentration in digesters results in that the acetate reaction is the primary producer of methane [21]. The methanogenic bacteria include *Methanobacterium, Methanobacillus, Methanococcus, *and* Methanosarcina.* Methanogens can also be divided into two groups: acetate and H_2_/CO_2_ consumers. Also, *Methanosaeta *is considered to be important in AD as both acetate and H_2_/CO_2_ consumers [21, 23]:

(8) $$\begin{array}{c} \text{C}{\text{H}}_{3}\text{COOH}⟶\text{C}{\text{H}}_{4}+\text{C}{\text{O}}_{2} \end{array}$$

Carbon dioxide reduction is as follows:

(9) $$\begin{array}{c} \text{C}{\text{O}}_{2}+4{\text{H}}_{2}⟶\text{C}{\text{H}}_{4}+2{\text{H}}_{2}\text{O} \end{array}$$

The challenges facing anaerobic digestion such as low methane yield and process instability, preventing this technology from being widely applied. A wide variety of inhibitory substances are the primary cause of anaerobic digester upset or failure since they are present in substantial concentrations in wastes. Considerable research efforts have been made to identify the mechanism and the controlling factors of inhibition. The methane forming bacteria is strictly anaerobic and even small quantities of oxygen are harmful to them [5]. Nitrogen is an essential nutrient for anaerobic organisms [24]. The ammonia concentration must be maintained in excess of at least 40–70 mg N L^−1^ to prevent reduction of biomass activity [25]. And high ammonia concentrations may lead to inhibition of the anaerobic digestion process [26–28].

However, improved reactor configuration can reduce the space instability as soon as possible. For example, the compartmentalized anaerobic reactor (CAR) [29] was separated into a distribution zone, a reaction zone, and a separation zone by adding three inners, which seem to keep the space stability. As a result, the CAR displays a great potential for its application.

## 4. Process Parameters of Anaerobic Bioreactor

Degradation of unwanted components/contaminants in the anaerobic treatment depends on several parameters. The main parameters are related to reactor operating conditions (temperature, pH, organic loading rate (OLR), HRT, SRT, and upflow velocity) and influent characteristics such as particle size distribution. These parameters and their effects are discussed in the following paragraphs.

### 4.1. Temperature

Temperature an important physical characteristic that affects the acceptability of water as well as water chemistry and water treatment [30]. Anaerobic bacteria are classified into “temperature classes” on the basis of the optimum temperature; the mesophiles survive in mesophilic temperature around 30°C to 40°C, while thermophiles are considered the first microorganism existing at thermophilic temperature around 50°C to 65°C [31]. Temperature even affects all wastewater treatment processes to some degree [30], for example,

- biological waste treatment: cold water reduces the efficiency of high-rate trickling filters by approximately 30%; low temperature inhibits nitrification (by 75% from 30°C to 10°C) more than BOD (biological oxygen demand) removal;
- digestion: the minimum solid retention time varies from 2 days at 35°C to 10 days at 20°C; the heat requirement of the digester depends on outside temperature;
- microbial growth: temperature affected the richness and diversity of microbial populations [32].

Temperature affects particle removal through influencing the wastewater viscosity and conversion of organic matter [33]. Because water viscosity is physically coupled to temperature, changes in temperature can influence the activity of microscopic organisms through both physiological and physical means [34]. Increasing the wastewater temperature leads to enhancing mixing by reducing viscosity, more hydraulic turbulence in a reactor, enhanceing the sedimentation, and better entrapment and adsorption due to contact between sludge and solids, and more biogas will be produced [33]. A sudden temperature changes can lead to a change in the physical and chemical properties of the wastewater, which can considerably affect the design and operation of the treatment system [35].

#### 4.1.1. Low Temperatures

Generally temperature has a significant effect on the intracellular and extracellular environment of bacteria, and it also acts as an accelerator of the conversion processes. At low temperatures the startup period may take longer, but it can be successfully accomplished by inoculating the reactor with digested sludge. Anaerobic treatment of raw domestic sewage (COD = 500–700 g m^−3^) on different UASB reactors can be accomplished at 12–18°C (HRTs of 7–12 h with total COD and BOD removal efficiencies of 40–60% and 50–70%, resp.) [3]. And also the possibility of applying anaerobic digestion of dilute dairy waste water at 10°C at OLRs up to 2 kg COD m^−3^ d^−1^ can gain over 84% of OD removal efficiency [36].

#### 4.1.2. High Temperatures

The rates of reaction proceed much faster at higher temperatures, therefore producing more efficient operation and smaller tank sizes. And also treatment proceeds much more rapidly at thermophilic temperatures (around 50°C to 65°C), and the digestion under high temperature conditions offers many advantages such as higher metabolic rates, consequently higher specific growth rates, but frequently also higher death rates as compared to mesophilic bacteria, but also the additional heat required to maintain such temperatures may offset the advantage obtained. Then, most treatment systems are designed to operate in the mesophilic range or lower [5, 37, 38].

### 4.2. pH

pH is an expression of the intensity of the basic or acid condition of a liquid, a measure of the acidity of a solution. Commonly, methanogens in wastewater treatment systems are most active in the neutral pH range (7.0) [39]. The concentration range suitable for most organisms is 6.0–9.0 [30]; beyond this range, digestion can proceed, but with less efficiency. The biomass inhibited at pH 9 was able to regain activities after adjusting the pH to neutrality, but that inhibited at pH 5 was not [40]. At acidic conditions produced can become quite toxic to the methane bacteria. For this reason, it is important that the pH is not allowed to drop below 6.2 for a significant period of time. Because this parameter is very important, thus the system needs to control the pH. When the methane gas production stabilizes, the pH remains between 7.2 and 8.2 [19].

McCarty [5] reported that an optimum pH range of anaerobic treatment is about 7.0 to 7.2, but it can proceed quite well with a pH varying from about 6.6 to 7.6.

### 4.3. Hydraulic Retention Time (HRT)

HRT also known as hydraulic residence time is a measure of the average length of time that a soluble compound remains in a constructed bioreactor. Hydraulic retention time is the volume of the aeration tank divided by the influent flow rate:

(10) $$\begin{array}{c} \text{HRT}\;\left[\text{d}\right]=\frac{\text{Volume}\;\text{of}\;\text{aeration}\;\text{tank}\;\left[{\text{m}}^{3}\right]}{\text{Influent}\;\text{flow}\;\text{rate}\;\left[{\text{m}}^{3}/\text{d}\right]}=\frac{V}{Q}, \end{array}$$

where HRT is hydraulic retention time (d) and usually expressed in hours (or sometimes days), the *V* is the volume of aeration tank or reactor volume (m^3^), and *Q* is the influent flow rate (m^3^/d).

Generally HRT is a good operational parameter that is easy to control and also a macroconceptual time for the organic material to stay in the reactor. In bioreaction engineering studies, the reverse of HRT is defined as dilution rate, for which if it is bigger than the growth rate of microbial cells in the reactor, the microbe will be washed out, and otherwise the microbe will be accumulated in the reactor. Either of these situations may result in the breakdown of the biological process happening in the reactor.

### 4.4. Organic Loading Rate (OLR)

At the industrial scale a range of high-rate anaerobic fluidized-bed (AFB) reactors such as upflow anaerobic sludge blanket (UASB), upflow-staged sludge bed (USSB), expanded granular sludge bed (EGSB), internal circulation (IC), and inverse anaerobic fluidized bed (IAFB) reactors can bear very high loading rates, up to 40 kg COD/(m^3^·d) [41]. The organic loading rate (OLR) and volumetric biogas production (VBP) of the spiral automatic circulation (SPAC) reactor in our laboratory could reach up to 306 kg COD/(m^3^·d) [41–43]. Several authors reported that up to a certain limit, the treatment efficiency of complex wastewaters, for example, potato maize, slaughterhouse, in high rate anaerobic reactors increases with increase in OLR. A further increase in OLR will lead to operational problems like sludge bed flotation and excessive foaming at the gas-liquid interface in the gas-liquid-solid (GLS) separator, as well as accumulation of undigested ingredients. As a result, the treatment efficiency deteriorates [44, 45]. Also accumulation of biogas in the sludge bed was noticed, forming stable gas pockets that lead to incidental lifting of parts of the bed and a pulse-like eruption of the gas from this zone [45, 46]. The OLR can be varied by changing the influent concentration and by changing the flow rate. Thus, implies changing the HRT and by changing the flow rate, under these conditions OLR can be expressed in the following form:

(11) $$\begin{array}{c} \text{OLR}=\frac{\left(Q\times \text{COD}\right)}{V}, \end{array}$$

where OLR is organic loading rate (kg COD/m^3^·d), *Q* is flow rate (m^3^/d), COD is chemical oxygen demand (kg COD/m^3^), and *V* is reactor volume (m^3^). By using (10), the OLR can be simplified:

(12) $$\begin{array}{c} \text{OLR}=\frac{\text{COD}}{\text{HRT}}. \end{array}$$

When the solids removal efficiency in upflow reactors is related to the OLR, it becomes crucial to distinguish between these parameters. For this reason, OLR is an inadequate design parameter to assure good performance of anaerobic reactors.

### 4.5. Sludge Retention Time (SRT)

SRT is known to be the key parameter affecting biochemical and physical properties of sludge [47]. The success of UASB reactors is mainly dependent on the sludge retention time (SRT) [48], which is the key factor determining the ultimate amount of hydrolysis and methanogenesis in a UASB system at certain temperature conditions [49]. The SRT should be long enough to provide sufficient methanogenic activity at the prevailing conditions. The SRT is determined by the loading rate, the fraction of suspended solid (SS) in the influent, the removal of SS in the sludge bed, and the characteristics of the SS (biodegradability, composition, etc.) [3].

Methanogenesis starts at SRT between 5 and 15 days at 25°C and between 30 and 50 days at 15°C, the maximum methanogenesis found at 25°C amounted to 51% and 25% at 15°C. Maximum hydrolysis occurs at 75 days SRT and amounted to 50% at 25°C and 24% at 15°C [47]. The SRT and temperature have a significant influence on the hydrolysis of proteins, carbohydrates, and lipids. The most substantial portion of the digestion of proteins, carbohydrates, and lipids occurs within the first 15 and 10 days at process temperatures of 25°C and 35°C, respectively [50].

### 4.6. Upflow Velocity

The upflow velocity is one of the main factors affecting the efficiency of upflow reactors. An increase in upflow velocity from 1.6 to 3.2 m/h resulted in a relatively small loss in SS removal efficiency, from 55% to nearly 50%, which indicates the role of adsorption and entrapment [51].

### 4.7. Particle Size Distribution

The particle-size distribution (PSD) of a powder, granular material, or particles dispersed in fluid is a list of values or a mathematical function that defines the relative amount, typically by mass, of particles present according to size. The effluent quality from classical filters is highly related to the specific size of the filtering media. Most studies indicate that smaller media size gives more efficient removal [52].

## 5. Inner Components of Anaerobic Bioreactor

The inner components play an important role in enhancing OLR of the reactor, help in improving the quality of fluidization, separate the gas bubble from the sludge granules, and therefore enhance the treatment efficiency and so on. The inner components in a biological fluidized bed reactor can be divided into the transverse inner components, longitudinal inner components, and biofilm-packing material.

### 5.1. Transverse Inner Components

**Figure 3:**
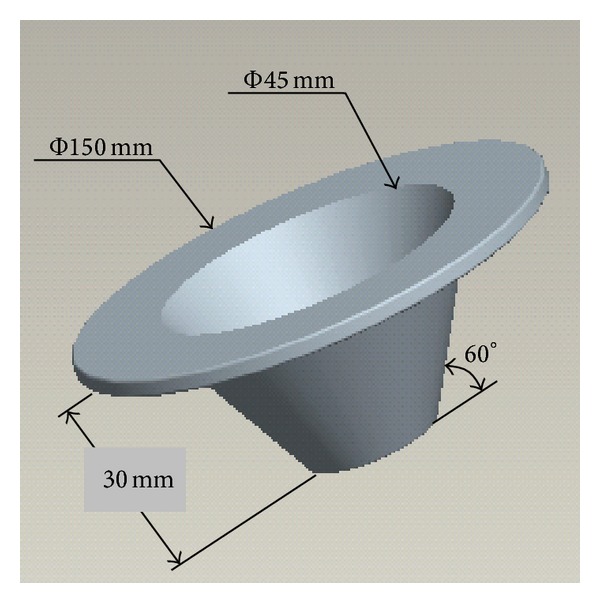
Structural sketch diagram of funnel-shape inner component.

The most significant function of transverse inner components is (1) to keep the sludge in AB effectively and (2) to improve the quality of fluidization, break bubbles, and enhanced mass transfer significantly. To keep sludge effectively, following a three-phase separator developed in the early 1990s by Lettinga et al. [53], many investigators set inner components in the reactor by applying the three-phase separation principle. Chelliapan et al. [54] set a 45° angle of the three-phase separator baffle in the outlet of upflow anaerobic a multistage reactor (UASR) and held the granular sludge efficientively (5850 g VSS·m^−3^). In order to reduce the short flow, improve the flow pattern, and enhance mass transfer, many researchers set different inner components in AB.  Figure 3 shows that the funnel-shaped diversion components were located in the top of the reactor resulting in a 15% increase in the volumetric oxygen transfer coefficient, and liquid mixing time is reduced by 10% to 25% [55].

**Figure 4:**
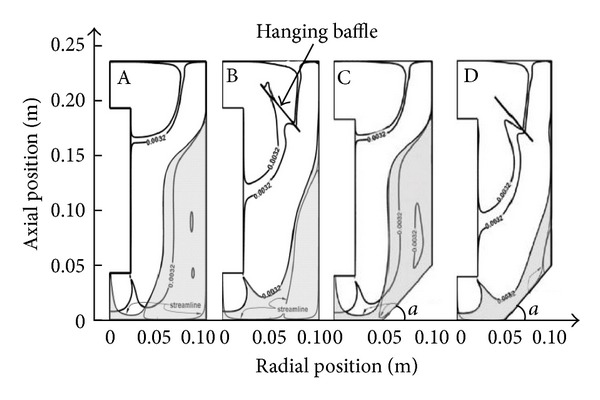
Comparison of fluidization quality for different conical bottom digesters with and without hanging inner component.

Karim et al. [56] studied the effect of bottom configuration and a hanging baffle on the mixing inside a gas-lift digester filled with non-Newtonian sludge (Figure 4 and Table 2). Their results showed that to change the configuration of the bottom of the reactor (Figures 4(a) and 4(c)), the figures demonstrate that the conical bottoms resulted in only slight improvement, but Figures 4(b) and 4(d) show additional suspended inner component to permit fluid flow to the inner wall of the reactor. Under the action of an inverted conical head, the fluid flows within the draft tube, thereby constituting internal circulation, and reducing short-stream and the dead zone.

The data in Table 2 shows that the change in digester bottom configuration resulted in about 2–4% reduction in the poorly mixed zone. However, the introduction of a hanging baffle reduced the percentage of poorly mixed zones by 12%, 16%, and 18% in the case of flat, 25° and 45° bottom digesters, respectively. Therefore, it is clear that a combination of a hopper bottom and a hanging baffle would significantly improve the mixing efficacy inside gas-lift digesters.

### 5.2. Longitudinal Inner Components

**Figure 5:**
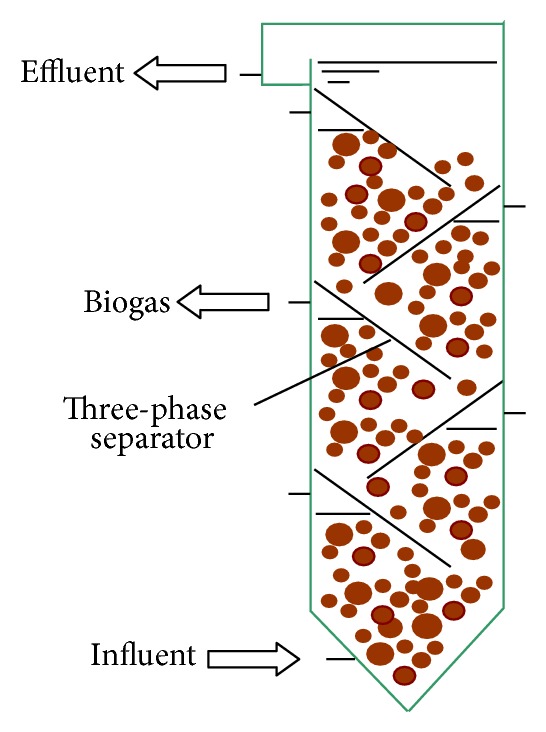
Structural and setup sketch of 3-phase separator baffle in USSB.

Recent research at home and abroad shows that the future development trend of longitudinal inner components is organic integration of the components within the multigroup, in order to optimize the flow field and reduce the intermediate product inhibition. Van Lier et al. [57] set a number of three-phase separators baffle in the sludge bed of an upflow staged sludge bed (USSB) reactor (Figure 5). Its performance compared to that of an upflow anaerobic sludge bed (UASB) reactor operating at the same operational conditions, and the reaction zone in (USSB) is divided into several compartments, and each compartment can collect produced gas [58, 59].

**Figure 6:**
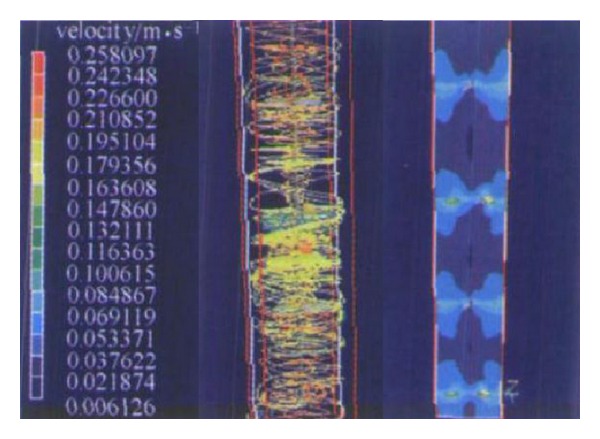
Simulation of flow field in SUFR.

Guyuan et al. and Ji et al. [60, 61] studied the performance of the inner components having a certain inclination multilayer baffle deflector driven in the spiral upflow reactor (SUFR) by software simulation (Figure 6). Hong-lin et al. [62–64] reported that the plurality of sets of helical blades combined inner longitudinal component used in fluidized bed reactor to enhance the reactor coagulation, granulation, and biological degradation.

### 5.3. Biofilm-Packing Material

In 1978, Weber Jr. et al. [65] placed in a fluidized bed reactor dosing activated carbon particles and explored the inner structures of biofilm as the first of its kind. Granular activated carbon dosing has three functions to provide huge growth of microbial attachment specific surface area, enhanced biosorption, and biodegradation of synergies, greatly improve the matrix of the particle surface and oxygen concentration, effectively enhance the performance of microbial oxidation, effectively relieve hydraulic shearing action, and maintain a high concentration of microorganisms. In1980s, fixed filler was placed by McCarty and Smith [66] in the bioreactor as the inner component. High concentrations of anaerobic activated sludge was achieved, and thus anaerobic filter (AF) was developed [67]. Since then, many researchers added activated carbons [68], sand [69] calcium alginate [70], and porous polymer carrier [71] and so on as inner component in AF, and achieved some remarkable efficiency.

## 6. Super-High-Rate Anaerobic Bioreactor (SAB)

**Figure 7:**
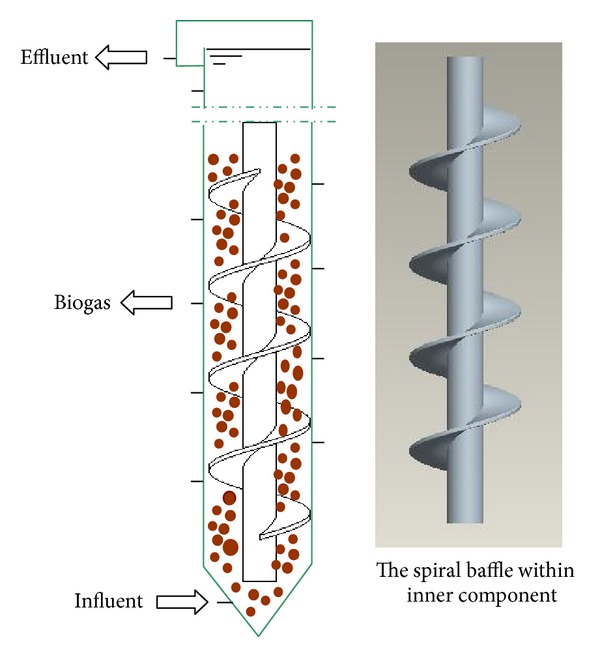
Structural and setup sketch of spiral baffle.

Under high load conditions, the biogas produced in the anaerobic reactor does not escape granular sludge in a timely manner, can reduce the particle density, and increase bed slugging. In laboratory-scale biological fluidized bed, slugging cause paralysis of the mechanical operation of the reactor. In response to this situation, Zheng's research group has developed a new spiral automatic circulation (SPAC) anaerobic reactor [42] (Figure 7), after more than two years, OLR of SPAC can reach up to 300 Kg COD·m^−3^·d^−1,^ much higher than the performance level of the existing high-rate anaerobic reactor [43]. Its main advantages are to effectively eliminate the slugging phenomenon and ensure the smooth operation of the reactor by means of the reaction force of the spiral plate reactor.

## 7. Conclusion

(i) Anaerobic treatment is a proven way and efficient method to produce biogas (methane) that can be used for the production of renewable heat and power and a compost like output. Inhomogeneous system, time instability, and space instability are three main characteristics of anaerobic bioreactor (AB).

(ii) Anaerobic treatment efficiency has a deep effect by several parameters such as temperature, pH, OLR, SRT, HRT, upflow velocity, and size distribution, cause of anaerobic reactor operates under inhomogeneous system (Gas-liquid-solid). Therefore anaerobic treatment needs especial kind of setting because anaerobic processes successful depends on bacteria living and growth inside the reactor.

(iii) The reactor inner components play an important role in enhancing the treatment efficiency, and the most significant functions of inner components are (1) effective to improve the retention sludge reactor capacity and (2) significantly improve the quality of fluidization, broken bubbles, and enhanced mass transfer. And they can be divided into the transverse inner components, longitudinal inner components, and biofilm-packing material.

## Figures and Tables

**Table 1:** Advantages and disadvantages of anaerobic process [3, 15].

Advantages	Disadvantages
(i) Reduction of greenhouse gas emissions through methane recovery. (ii) High treatment efficiency for biodegradable sludge. (iii) Production of methane gas (potential source of fuel). (iv) A high degree of waste stabilization is possible. (v) Simplicity. (vi) Flexibility: anaerobic treatment can easily be applied on either a very large or a very small scale. (vii) Space saving (higher loading rates require smaller reactor volumes thereby saving on disposal cost). (viii) Less requirement of energy and oxygen. (ix) Inoffensive residual sludge may be used as soil conditioner. (x) Low nutrients and chemicals requirement.	(i) Long recovery time: it may take longer time for the system to return to normal operating conditions if shock loading happens. (ii) Low pathogen and nutrient removal. (iii) Long startup. (iv) Possible bad odors. (v) High sensitivity of methanogenic bacteria to a large number of chemical compounds. (vi) Small- and middle-scale anaerobic technology for the treatment of solid waste in middle- and low-income countries is still relatively new.

**Table 2:** Comparison of fluidization quality for different conical bottom digesters with and without hanging inner component.

Bottom configuration	The short flow area of the total volume (%)	
	No inner components	Inner components
Flat-bottomed head	33.6	21.4
Conical head (*α* = 25°)	31.9	15.8
Conical head (*α* = 45°)	29.6	11.7
